# A novel approach for the isolation and long-term expansion of pure satellite cells based on ice-cold treatment

**DOI:** 10.1186/s13395-021-00261-w

**Published:** 2021-03-17

**Authors:** Anna Benedetti, Gianluca Cera, Daniele De Meo, Ciro Villani, Marina Bouche, Biliana Lozanoska-Ochser

**Affiliations:** 1 Department of Anatomical, Histological, Forensic and Orthopedic Sciences, Section of Histology and Embryology, Sapienza University of Rome, Rome, Italy; 2 Department of Anatomical, Histological, Forensic and Orthopedic Sciences, Section of Orthopedics, Sapienza University of Rome, Rome, Italy; 3 Department of Orthopaedics and Traumatology, Policlinico Umberto I, Rome, Italy

**Keywords:** Satellite cell isolation, Satellite cells in vitro expansion, Skeletal muscle regeneration

## Abstract

Satellite cells (SCs) are muscle stem cells capable of regenerating injured muscle. The study of their functional potential depends on the availability of methods for the isolation and expansion of pure SCs with preserved myogenic properties after serial passages in vitro. Here, we describe the ice-cold treatment (ICT) method, which is a simple, economical, and efficient method for the isolation and in vitro expansion of highly pure mouse and human SCs. It involves a brief (15–30 min) incubation on ice (0 °C) of a dish containing a heterogeneous mix of adherent muscle mononuclear cells, which leads to the detachment of only the SCs, and gives rise to cultures of superior purity compared to other commonly used isolation methods. The ICT method doubles up as a gentle passaging technique, allowing SC expansion over extended periods of time without compromising their proliferation and differentiation potential. Moreover, SCs isolated and expanded using the ICT method are capable of regenerating injured muscle in vivo. The ICT method involves minimal cell manipulation, does not require any expertise or expensive reagents, it is fast, and highly reproducible, and greatly reduces the number of animals or human biopsies required in order to obtain sufficient number of SCs. The cost-effectiveness, accessibility, and technical simplicity of this method, as well as its remarkable efficiency, will no doubt accelerate SC basic and translational research bringing their therapeutic use closer to the clinic.

**Supplementary Information:**

The online version contains supplementary material available at 10.1186/s13395-021-00261-w.

## Background

The muscle is endowed with an exceptional regenerative ability primarily due to a resident population of stem cells called satellite cells (SCs) [1–3]. Ensconced between the basal lamina and the plasma membrane of muscle fibers, SCs respond to injury or various stress stimuli by becoming activated, and undergoing proliferation, self-renewal, and differentiation to form new myofibers [4, 5]. As SCs become activated, a proportion of them identified as Pax7^+^MyoD^-^ replenish the stem cell pool, while others acquire the expression of MyoD (Pax7^+^MyoD^+^), differentiate into myoblasts and enter the myogenic program. After several rounds of division myoblasts give rise to Myogenin^+^ myocytes, which fuse together to form new myofibers [1, 4, 6–9].

A major stumbling block in the study of the functional potential of SCs has been the lack of isolation methods involving minimal cell manipulation and allowing the isolation and expansion of highly pure SCs with preserved myogenic properties after serial passages in vitro [10–12]. Moreover, the success of SC transplantation therapy depends on having an efficient method to isolate and expand these cells in vitro in undifferentiated state and in sufficient numbers [13].

Presently, there are three main methods commonly used for the isolation of SCs: the pre-plating method, fluorescence activated cell sorting (FACS), and magnetic bead isolation method.

The pre-plating method is based on the differing adhesive properties of muscle cells, with SCs being the least adherent. Following enzymatic digestion, a heterogeneous mix of skeletal muscle cells is plated onto uncoated culture dishes and after a 1–24-h incubation at 37 ^○^C, the non-adherent cells are collected and plated onto new collagen coated dishes [10–12, 14]. The resulting cell culture contains both SCs and fibroblasts in variable proportion. To improve SC purity, the pre-plating step can be repeated every 24 h over 6 days [10]. Although cheap and straightforward to perform, this method’s main disadvantage is that it is time consuming and gives rise to cultures of variable purity, with fibroblast contamination and overgrowth by day 7 of culture, leading to early senescence and detachment of myotubes [10]. A recently described version of the pre-plating method introduces a re-plating step whereby after a 2-day expansion, the adhered cells are detached with trypsin and replated onto Matrigel coated dishes, giving rise to SC cultures of much improved purity [15].

The FACS sorting method sorts muscle mononuclear cells pre-labelled with SC specific antibodies. Following digestion of muscle with various enzymes the resulting mixture of cells is labelled with specific antibodies to facilitate the identification of SCs, which are then sorted using a FACS sorter instrument [16–21]. At present, the FACS sorting method represents the gold standard for the isolation and study of SCs. Nevertheless, there are several disadvantages to this method including high cost and the requirement for a FACS sorter instrument. Moreover, this method is time consuming, requires expertise to perform and cell purity can be variable. The cell labelling step followed by the sorting procedure can potentially stress or damage the cells, decrease their viability, or alter their functional properties in vitro [12]. The third method is based on magnetic cell separation (MACS) and uses magnetic columns and SC specific magnetic bead kits [22]. It is based on negative selection of SCs by magnetically labelling and removing other cell lineages. Because this method assumes that all the other cell types are successfully removed from the muscle cell preparation, it is less precise than the FACS sorting method. This method is expensive to perform, time consuming, and stressful for the cells. As for the other two methods, cell purity is variable and often the SC cultures become overgrown by fibroblasts by day 7 [10, 12].

There is therefore a need for new and improved methods for the isolation, expansion and culture of SCs. Here, we describe a simple, inexpensive, and efficient method for the isolation of highly pure mouse and human SCs that can be serially expanded in vitro to obtain sufficient number of SCs with preserved proliferation potential, capable of regenerating injured muscle in vivo.

## Methods

### Mice

C57BL/10ScSn-*Dmd*^*mdx*^, C57BL/10ScSn, and C57BL/6J mice were purchased from the Jackson laboratory (Bar Harbor, ME, USA). Both male and female mice were used. The mice were housed in the Histology Department–accredited animal facility at the University of Sapienza. All the procedures were approved by the Italian Ministry for Health and were conducted according to the EU regulations and the Italian Law on Animal Research.

### Human muscle sourcing

Muscle biopsies (gluteus maximus) were obtained from patients (8 males and 7 females, age range 50–90 years) undergoing surgery at the Department of Orthopaedics and Traumatology, Umberto I Hospital in Rome, Italy. According to the Italian law, the authors are not required to ask for approval from an institutional review board or ethical committee for this type of study. In any case, all patients gave their approval to undergo intraoperative muscle biopsy and to publish the clinical and laboratory data obtained.

### Satellite cell isolation with the ice-cold treatment method

SCs were isolated from hind-limb muscles of 4–8-week-old mice or from human biopsies. Muscles were dissected with scissors and finely diced with a scalpel in a dish containing DMEM (Sigma-Aldrich, St. Louis, MO, USA, D5671). This was followed by enzymatic digestion with 10 ml/g of muscle of Collagenase type II (Sigma-Aldrich, SCR103) at a concentration of 0.4 mg/ml in PBS (Sigma-Aldrich), for 45 min in a water bath at 37 °C with agitation. Digestion was blocked with DMEM 10% FBS and after centrifuging the muscle preparation and removing the supernatant, a second digestion was performed with 10 ml/g of muscle of Collagenase/Dispase at a concentration of 1 mg/ml (Roche, Basel, CH, 11097113001) in PBS Calcium-Magnesium free (Sigma-Aldrich), for 30 min at 37 °C in a water bath with agitation. The digested muscle was then passed first through a 70-μm cell strainer followed by 40-μm cell strainer to obtain single cell suspension. Next, the cells were centrifuged, resuspended in DMEM 10% FBS (Sigma-Aldrich, F2442), counted and plated at 2 × 10^6^ cells/100 mm dish (uncoated) (Corning, NY, USA, 430167), and incubated at 37 °C for 1 h. Non-adherent cells were collected, centrifuged, and the cell pellet was resuspended in DMEM 10% FBS, plated again, and incubated for another 1 h at 37 °C. After the second pre-plating, non-adherent cells were collected, centrifuged, counted, resuspended in SC Growth Medium (GM) DMEM, 20% Horse Serum (Thermo Fisher Scientific, Waltham, MA, USA, 26050088), 3% Chicken Embryo Extract (Seralab, CE-650-J), and plated into 100 mm dishes coated with 0.1% gelatin (Stem Cell Technologies, Vancouver, BC, CAN, 07903), at 10^6^ cells/dish. The next day, the dishes containing a heterogeneous mix of adhered muscle cells were washed 3 times with PBS, and 10 ml of ice-cold PBS was added into each dish. The dishes were then placed on ice (0 °C) for 15–30 min with occasional gentle manual shaking (swirling motion). The detached cells were collected, centrifuged, resuspended in GM, and plated into 0.1% gelatin-coated 35-mm dishes (Corning, 353001) at a density of 10^3^ cells/dish. To induce differentiation, proliferating cells (day 3 after adding GM) were cultured in differentiating medium (DM) containing DMEM 5% Horse Serum, 1% Chicken Embryo Extract.

### Satellite cell isolation with magnetic bead labelling

Mouse SC isolation by magnetic bead labelling was performed by using a SC Isolation Kit (Miltenyi Biotech, Bergisch Gladbach, DE, REF: 130-104-268) as previously described [23]. Briefly, minced muscle was digested as described above. The digested muscle was passed through 70 μm and 40 μm cell strainers, and the resulting single cell suspension was centrifuged, resuspended in 80 μl buffer (PBS pH 7.2, 0.5% FBS, 2 mM EDTA), and incubated with 20 μl of Satellite Cell Isolation Kit per gram of muscle, for 15 min at 4 °C. Next, the cell suspension was passed through a LS column (Miltenyi Biotech, 130-042-401) placed in a magnetic field of a MACS Separator (Miltenyi Biotech). Unlabeled SCs were collected in the flow-through, counted, washed, resuspended in growth medium (GM), and plated into 35-mm dishes at a density of 10^3^ cells/dish.

Mouse and human SCs were cultured either in GM containing DMEM, 20% Horse Serum (Sigma-Aldrich), 3% Chicken Embryo Extract, or in DM containing DMEM 5% Horse Serum, 1% Chicken Embryo Extract.

### SC detachment with trypsin

SCs were rinsed once with PBS and then incubated with trypsin-EDTA solution (Sigma-Aldrich, T3924) for 5 min at 37 °C. SCs were then collected, centrifuged, resuspended in GM, and plated at a density of 10^3^ cells/dish.

### SC transplantation

Acute muscle injury was induced the day before SC transplantation. To induce muscle injury tibialis muscles were injected with 0.01 ml of Cardiotoxin from Naja Pallida (10 μM) (Latoxan ZA, Les Auréats, Fr), using a 30 Gauge micro-syringe [23–26].

For cell transplantation, 15,000 SCs were resuspended in 20 μl of DMEM 2% FBS (Sigma-Aldrich) and injected into the TA muscle of one leg with a single injection by using a 30 Gauge micro-syringe. Contralateral TA muscle was injected with only PBS and used as control.

### Immunofluorescence and microscopy

For immunofluorescence analysis cultured SCs were fixed in PFA 4% for 10 min RT, permeabilized in cold methanol at – 20 °C for 6 min, blocked in 5% Goat Serum in PBS for 30 min RT, and incubated overnight at 4 °C in 4% BSA in PBS with the following primary antibodies: mouse anti-Pax7-c (1:10 DSHB, Iowa City, IA, USA), rabbit anti-MyoD (1:50 Santa Cruz C20: sc-304, Dallas, TX, USA), mouse anti-myogenin (1:20, DSHB), mouse anti-Myosin Heavy Chain (1:20 DSHB), and mouse anti-desmin (1:20, DSHB). The next day, SCs were washed 3 times in PBS for 15 min, and then incubated with secondary antibodies goat anti-rabbit Alexa Fluor 488 (1:1000, Abcam) and goat anti-mouse Alexa Fluor 555 (1:1000, Thermo Fisher Scientific) diluted in 1% BSA in PBS, for 1 h RT. Nuclei were counterstained with Hoechst.

For immunofluorescence analysis of mdx TA muscle transplanted with WT SCs, 8-μm-thick muscle cryosections were fixed in 4% PFA for 10 min at room temperature (RT), and then permeabilized in cold methanol for 6 min at – 20 °C. Sections were then blocked in 5% Goat Serum (Sigma-Aldrich) in PBS for 30 min at RT. Next, sections were incubated with primary rabbit anti-dystrophin antibody (1:200, Abcam, Cambridge, UK) overnight at 4 °C. The next day, sections were washed and incubated with a secondary antibody goat anti rabbit Alexa Fluor 488 (1:1000 Abcam). Nuclei were counterstained with Hoechst.

Samples were analyzed under an epifluorescence Zeiss Axioskop 2 Plus microscope (Carl Zeiss, Oberkochen, DE).

Bright field images were acquired with an inverted phase-contrast microscope (Nikon Eclipse, TS100). Images were acquired with a Nikon DS-Fi2 camera and NIS Elements version 4.0 Imaging System.

### CFSE staining

Isolated SCs were stained with CFSE (ThermoFisher Scientific) at a concentration of 1 μM for 10 min at 37 °C in the dark prior to culture. After 4 days of culture, the cells were detached with Accutase Solution (Sigma-Aldrich). Samples were acquired with a CyAn ADP (DAKO) flow cytometer and acquired data were analyzed using FlowJo software version 10 (FlowJo LLC, Ashland, OR, USA).

### RNA isolation and quantitative real-time PCR

For RNA preparation, cells were lyzed with TRI reagent (Sigma-Aldrich) and processed as previously described [23]. Reverse transcription was performed with SensiFAST™ cDNA Synthesis Kit (Bioline, Memphis, TN, USA). Quantitative real-time PCR assays were performed according to the *MIQE* criteria, using SensiFAST™ SYBR No-ROX Kit (Bioline) following manufacturer’s protocol. All reactions were performed in duplicate. Data were collected and analyzed using ABI PRISM 7500 Sequence Detection System (Life Technologies, Carlsbad, CA, USA). Quantitative RT–PCR values were normalized to the expression of GAPDH mRNA. The relative gene expression level was calculated using the 2−ΔΔCT method and reported as mean fold change in gene expression.

The following primers were used for amplification: Pax7 (FW: 5′ GTCCCAGTCTTACTGCCCAC 3′, RV: 5′ TGTGGACAGGCTCACGTTTT 3′), Myogenin (FW: 5′ GCATGGAGTTCGGTCCCAA 3′, RV: 5′ TATCCTCCACCGTGATGCTG 3′), GAPDH (FW: 5′ ACCCAGAAGACTGTGGATGG 3′, RV: 5′ CACATTGGGGGTAGGAACAC 3′).

### Clonal myogenicity assay

For the clonal myogenicity assay, SCs were plated into 0.1% gelatin coated 96-well plates, (excluding the outer wells of the plate) at 1 cell per well, in growth medium. Colony formation and number of cells were assessed at 24, 48, and 72 h of culture.

### Statistical analysis

All statistical analyses were performed using GraphPad Prism software version 8 (La Jolla, CA, USA). Data are presented as mean ± SEM. Statistical significance was determined using unpaired 2-tailed Student’s *t* test with Welch’s correction for unequal variances. A *P* value of ≤ 0.05 was considered statistically significant.

## Results

### Isolation and characterisation of muscle SCs using the ice-cold treatment method

**Fig. 1:**
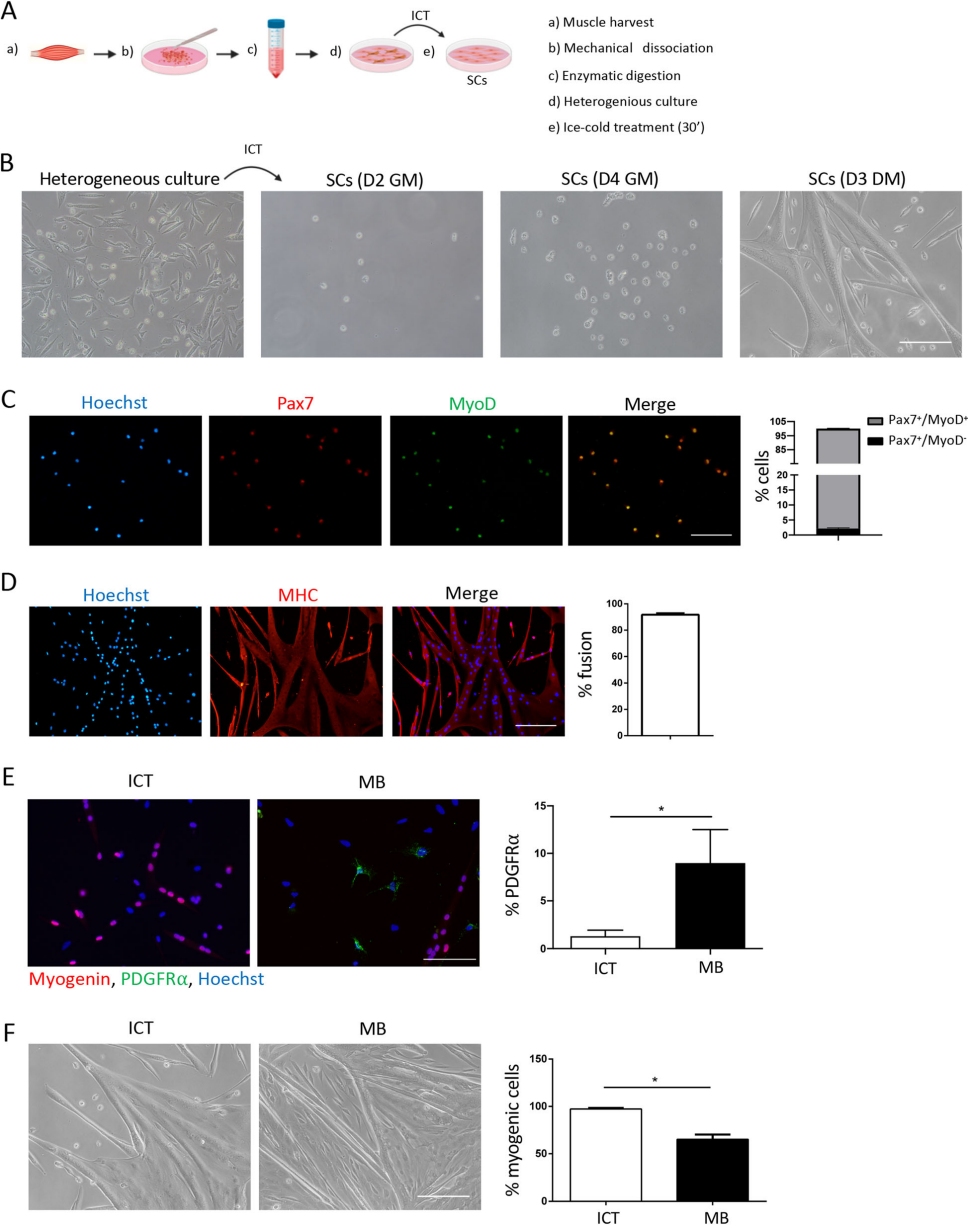
Isolation and characterization of muscle SCs using the ICT method. **a** Schematic representation of the ICT method. **b** Representative bright field images of the heterogeneous muscle mononuclear cell culture from which SCs were isolated by ICT, and representative images of the ICT-isolated SCs at D2 and D4 in growth medium (GM) and at D3 after adding differentiation medium (DM) (*n* = 15 independent experiments). **c** Representative immunofluorescence images of ICT-isolated SCs stained for Pax7 (red), MyoD (green), and nuclei (blue) and a graph showing the percentage of cells positive for Pax7 and/or MyoD at day 3 of culture in GM (*n* = 3 independent experiments). **d** Representative immunofluorescence images of ICT-isolated SCs stained for myosin heavy chain (MHC) (red) and nuclei (blue) and a graph showing percent fusion after differentiation (4 days in GM followed by 3 days in differentiating medium (DM)) (*n* = 3 independent experiments). **e** Representative immunofluorescence images of ICT-isolated SCs and MB-isolated SCs stained for Myogenin (red) PDGFR⍺ (green) and nuclei (blue) and a graph showing percent PDGFR⍺^+^ cells at day 5 of culture in GM. **f** Representative bright field images of ICT- and magnetic beads (MB)-isolated SCs and a quantification graph showing percentage of myogenic cells in ICT- and MB-isolated SCs after differentiation at day 7 of culture (*n* = 3 independent experiments). Non-myogenic cells were identified as Pax7^-^MyoD^-^nuclei outside the myotubes. Scale bar = 100 μm. Error bars represent mean ± sem. *p < 0.05 by Student’s *t* test.

Previous studies have demonstrated that cold temperature causes a reduction in cell adhesion, likely due to the downregulation of adhesion receptors [27, 28]. Compared to other cells, such as fibroblasts, which typically contaminate SC cultures, SC are considered to be less adherent [11, 14]. Taking this into consideration, as well as the notion that like all stem cells, SCs are sensitive to stress signals and are among the first muscle resident cells to respond to injury [4, 5], we hypothesized that subjecting a heterogeneous culture of muscle cells to a mild stress stimulus such as ice-cold temperature will lead to the detachment of only the SCs. To test this hypothesis, we obtained a mix of muscle cells following enzymatic digestion, and after 2 h of pre-plating on uncoated dishes, cultured them overnight on gelatin-coated dishes. The next day, after washing and removing the non-adhered cells and debris, we placed the dishes of heterogeneous muscle cells on ice for 30’ (Fig. 1a, b). This time point was chosen based on the purity and number of SCs obtained following 15, 30, 45, and 60’ on ice. Although the number of SCs obtained increased with longer incubations, the purity of SCs decreased from 100% at 15–30’ to 95 and 90% at 45 and 60’ on ice, respectively (data not shown). The ice-cold treatment (ICT) method can be used to harvest SCs from the original dish containing the heterogeneous muscle cells for at least the first 3 days of culture. Placing the heterogeneous muscle culture dish on ice for 30’ resulted in the detachment of only the SCs giving rise to a highly pure culture of SCs that proliferated and differentiated into myotubes upon culture in differentiating medium (Fig. 1b–d). Satellite cells isolated using the ICT method were 100% pure as determined by the expression of the SC markers Pax7 and MyoD (Fig. 1c). At day 3 of proliferation, 100% of the cells were positive for the satellite cell marker Pax7 and of these 97% were activated and expressed MyoD (Pax7^+^MyoD^+^) (Fig. 1c). To examine the myogenic capacity of ICT-isolated SCs we performed a clonal myogenicity assay, by plating a single cell per well and analyzing the formation of myogenic colonies. Satellite cells isolated with the ICT method displayed a similar clonal myogenicity of 40 % and a doubling time of 17 h to SCs isolated using the magnetic beads method (Figure S1A and B). The myogenic identity of the cultured cells was further confirmed by the expression of myosin heavy chain (MHC) and the formation of myotubes. After 3 days in differentiating medium the SCs differentiated into myoblasts that fused into MHC expressing myotubes with a fusion index of 90% (Fig. 1d). The purity of the isolated SCs at the beginning of culture, as well as the fusion index and number of myonuclei per myotubes were similar between SCs isolated with the ICT and magnetic cell separation (MACS) method (Figure S1C–E). However, by day 5–7, the cultures obtained with the MACS method became overgrown by non-myogenic cells such as PDGFRα^+^ fibroblasts, causing premature myotube detachment, whereas cultures obtained with the ICT method remained almost free of contaminating cells (Fig. 1e, f).

Overall, these results confirm our hypothesis that ICT leads to the preferential detachment of SCs and using this method allows the isolation of 99–100% pure SCs.

### The ICT approach can also be used for serial passaging and long-term expansion of SCs

**Fig. 2:**
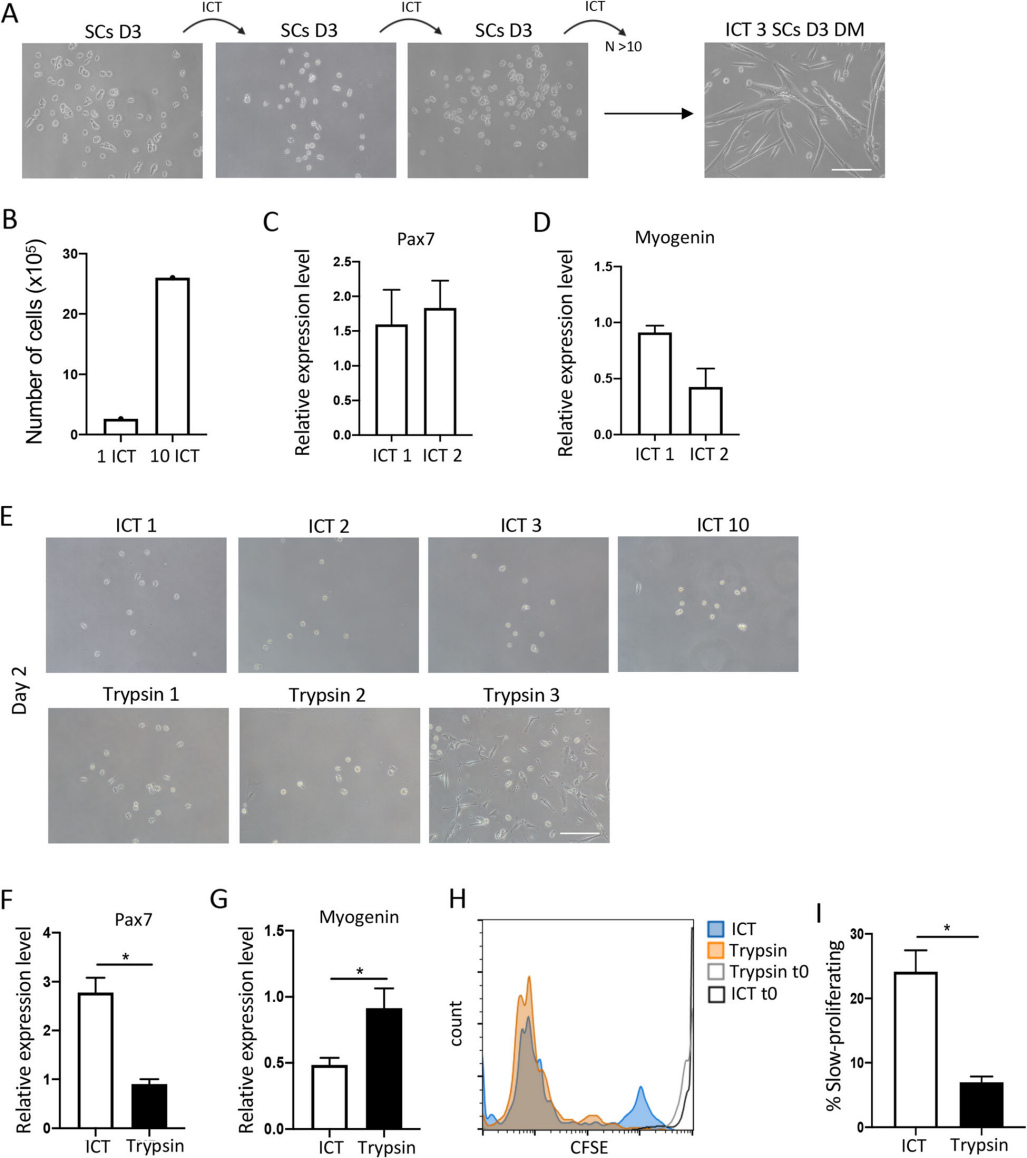
Serial expansion and long-term proliferation potential of SCs using the ICT method. **a** Representative bright field images of SCs at day 3 of culture in GM following ICT 1, ICT 2, and ICT 3, and differentiated cells at day 3 of culture in DM following ICT 3 (*n* = 10 independent experiments). **b** Total number of SCs at day 3 of culture in GM, after 1 and 10 ICTs (*n* = 10 independent experiments). **c** Pax7 gene expression in SCs at day 2 of culture in GM after ICT 1 and ICT 2 analyzed by quantitative real time PCR (*n* = 3 independent experiments). **d** Myogenin expression in SCs at day 5 of culture in GM after ICT1 and ICT 2 analyzed by quantitative real-time PCR (*n* = 3 independent experiments). **e** Representative images of SCs at day 2 of culture in GM, after 1–3 detachments with ICT (top panels) and trypsin (bottom panels). **f** Pax7 gene expression in SCs detached with ICT or trypsin, at day 2 of culture in GM, analyzed by quantitative real-time PCR (*n* = 3 independent experiments). **g** Myogenin gene expression in SCs detached with ICT or trypsin, at day 5 of culture in GM, analyzed by quantitative real time PCR (*n* = 3 independent experiments). **h** Representative overlay of histogram plots of CFSE labeled ICT and trypsin detached SCs at time 0 and day 4 of culture in GM. **i** Graph showing percent CFSE-low or slow-proliferating SCs after ICT or trypsin detachment, at day 4 of culture in GM (*n* = 3 independent experiments). Error bars represent mean ± sem, *p < 0.05, calculated by Student’s *t* test.

A major obstacle in SC research has been that cultured SCs lose their proliferation potential after a couple of passages and begin to differentiate into myotubes thereby limiting the number of SCs that can be serially expanded in vitro. Loss of differentiation potential has also been noted with increasing number of passages [10–13, 29]. We reasoned that, since the ICT leads to SC detachment, it can also be used to passage growing cultures of proliferating SCs. Indeed, placing the dishes of proliferating SCs on ice for 30 min led to the detachment of around 30% of the proliferating cells and we were able to serially passage and expand the proliferating SCs without compromising their proliferation and differentiation capacity (Fig. 2a). We used the ICT approach on proliferating SCs and then successively on each established culture of proliferating SCs (for more than 10 passages). This approach yielded on average 2.5 × 10^6^ cells/g of muscle (Fig. 2b). The SCs passaged using the ICT method did not lose their proliferation and differentiation potential and displayed a minimally altered Pax7 and myogenin gene expression (Fig. 2c, d). While ICT-passaged SCs retained their proliferation and differentiation capacity even after 10 passages, SCs passaged with the most commonly used passaging reagent trypsin, lost the ability to form new myogenic colonies after just 2 passages and instead begun to differentiate into myotubes (Fig. 2e–g). Similar to SCs detached by trypsin, SCs detached using a gentler detachment solution such as Accutase, exhibited accelerated differentiation after more than 3 passages (data not shown).

Previous studies have demonstrated that based on their rate of proliferation, SCs can be divided into two subpopulations: fast dividing and slow dividing [30]. The slow-dividing are a subset of SC that have been shown to retain stemness and long-term self-renewal ability [31]. Unlike the fast-dividing SCs, which have a limited ability to form secondary myogenic colonies after passage and instead undergo differentiation, the slow dividing SCs form secondary myogenic colonies when passaged [30, 31]. To examine the prevalence of fast and slow dividing SCs within our population, we labelled the ICT-passaged SCs with CFSE prior to plating and analyzed the rate of proliferation 3 days later. We compared the proliferation rate of ICT-isolated and passaged SCs with that of SCs isolated using magnetic beads and passaged with trypsin. In line with previous observations, we found that most of the activated SCs isolated using the commercial kit and passaged with trypsin were fast-dividing cells (CFSE^lo^), with slow-dividing cells (CFSE^hi^) representing less than 10% of the total. Interestingly, while similarly heterogeneous, the SCs isolated and passaged using the ICT method were enriched in the slow dividing SC population (Fig. 2h, i).

Next, we examined the longevity of differentiated ICT-isolated SCs in culture. The highly pure cultures of SCs isolated using the ICT method gave rise to myotubes that could be maintained in culture for up to 1 month, compared to just 7 days when isolated using the magnetic beads (Figure S2A–C) or the pre-plating method (Figure S2D).

Overall, these data show that the ICT method can be used for the serial expansion of SCs with preserved proliferation and myogenic potential over an extended period of time compared to other methods.

To examine the potential of ICT-isolated and expanded SCs to regenerate injured muscle, we injected 15,000 SCs isolated from wild-type mice into the tibialis anterior of mdx mice (lacking dystrophin) CTX injured 24 h previously. SCs were either injected immediately after ICT isolation, or after 3-day in vitro expansion following ICT. Transplantation of both SCs immediately after ICT-isolation and after in vitro expansion contributed to the regeneration process to a similar extent as evidenced by the appearance of newly formed dystrophin-positive fibers (Figure S3A and B). There was no difference in the ability of transplanted ICT-isolated SCs and SCs isolated by magnetic beads to regenerate injured muscle (Figure S3C and D). These experiments confirmed that SCs isolated with the ICT method successfully engraft after transplantation and do not lose their potential to regenerate injured muscle after expansion in vitro.

### Efficient isolation, serial expansion and long-term culture of human satellite cells with the ICT method

**Fig. 3:**
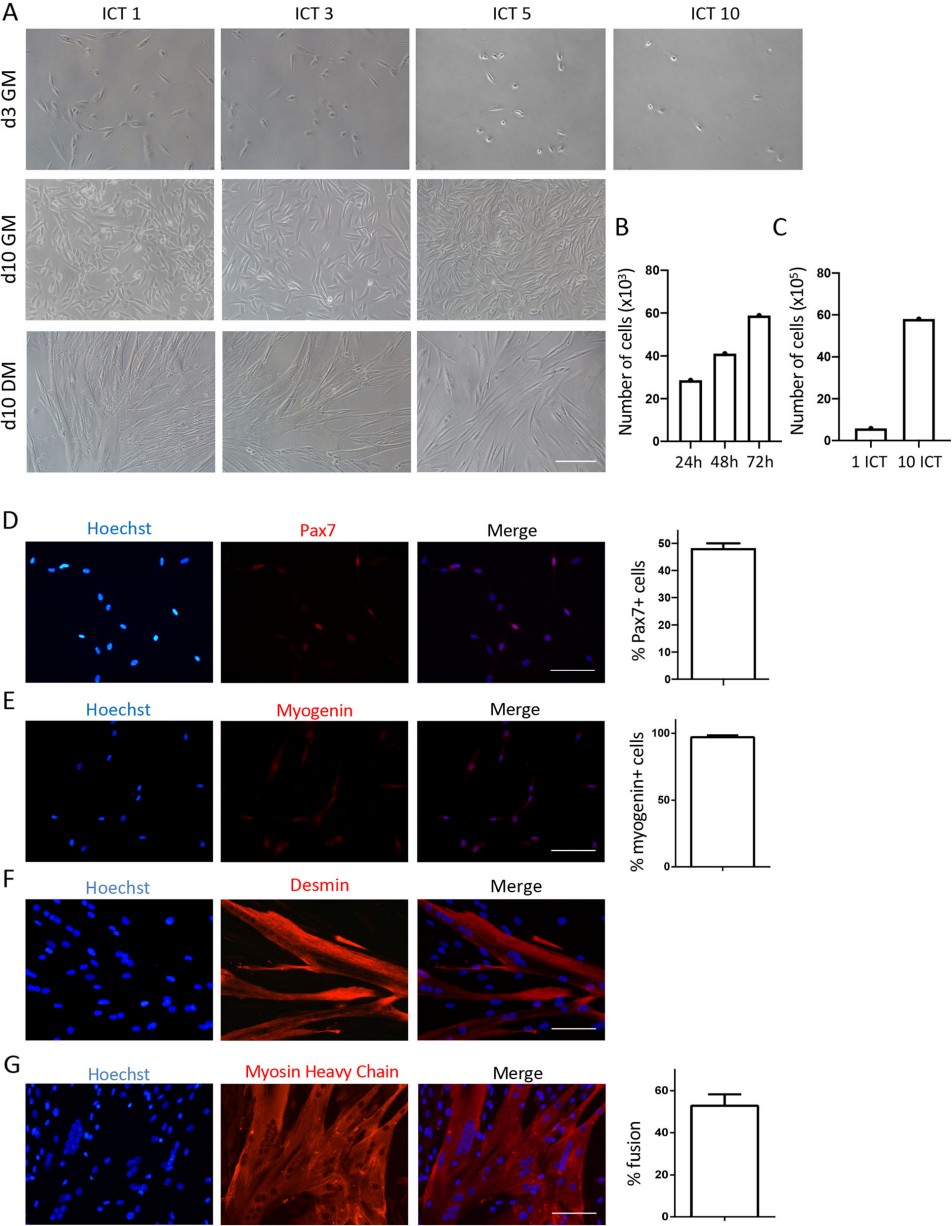
Isolation and in vitro expansion of human satellite cells using the ICT method. A. Representative bright field images of ICT- isolated human SCs following 1, 3, 5, and 10 ICTs, at day 3 and 10 of culture in GM, and at day 10 of culture in DM (*n* = 15 independent experiments). **b** Number of human SCs at 24, 48, and 72 h following ICT 1. **c** Total number of human SCs at day 3 of culture in GM after 1 and 10 ICTs. **d** Representative immunofluorescence images of human SCs stained for Pax7 (red) and nuclei (blue). Graph shows percentage of cells positive for Pax7 at day 2 of culture in GM. **e** Representative immunofluorescence images of human SCs stained for myogenin (red) and nuclei (blue). Graph shows percentage of cells positive for myogenin at day 5 of culture in GM. **f** Representative immunofluorescence images of human SCs stained for desmin (red) and nuclei (blue) after differentiation (10 days in GM + 5 days in DM). **g** Representative immunofluorescence images of human SCs stained for myosin heavy chain (MHC) (red) and nuclei(blue). Graph shows percent fusion after differentiation (10 days GM + 10 days DM). (*n* = 3 independent experiments, 10 images analyzed per experiment) Scale bar = 100 μm. Error bars represent mean ± sem

The study of human SCs (hSCs) has generally lagged behind that of mouse SCs due to the difficulties associated with obtaining muscle tissue, as well as the lack of methods for the isolation of pure hSCs that can be expanded in vitro without altering their myogenic potential [13]. Having demonstrated the remarkable efficiency of the ICT method in the isolation of pure mouse SCs, we set to reproduce these findings using human muscle biopsies. We obtained gluteus maximus specimens from patients undergoing surgery, aged between 50 and 90 years. Using the same approach as described above, we consistently and reproducibly obtained a highly pure culture of hSCs (Fig. 3a, d–g) from a heterogeneous population of human muscle cells (Figure S4), that could be serially expanded for more than 10 passages (Fig. 3a). As previously reported [32], we found that hSCs proliferated slower than mouse SCs, with a doubling time of 46 h (Fig. 3b), reaching a peak around day 10 post isolation, and slowing down thereafter. On average we isolated around 20 × 10^3^ SCs/g of muscle. Using the ICT method, these hSCs could be expanded 300-fold over a period of 2 months to a final total of 6 × 10^6^/g of muscle (Fig. 3c). Previous studies have shown that hSCs rapidly downregulate Pax7 expression in culture [33]. In agreement with others [33], we found that the expression of Pax7 was variable and ranged between 45 and 50% at day 2 of culture in GM (Fig. 3d). Almost 100% of the hSCs were myogenin positive at day 5 of culture in GM, suggesting that most of them have activated their myogenic program (Fig. 3e). The myogenic purity of the hSC culture was further confirmed by desmin at day 5 (Fig. 3f) and MHC expression at day 10 after shifting to differentiating medium (Fig. 3g).

These data show that the ICT method performs equally well when used for the isolation of human SCs.

## Discussion

Over the past decade, a considerable progress has been made in the development of new methodologies for the isolation of SCs. Nevertheless, each of the available methods suffers from at least one disadvantage be it purity, cost, expertise, or a combination of these [11]. In this study, we describe a novel method for the isolation of pure mouse and human SCs, that is inexpensive, simple to perform, and reproducibly efficient. The ICT method takes advantage of the differing adhesive properties of muscle cells as well as the ability of SCs to rapidly respond to stress stimuli [5, 11, 14]. Thus, the combination of a mild cold-stress stimulus and cold-induced reduction in adhesion, leads to the detachment of only the SCs. Exposure of mammalian cells to cold stress can slow down the progression through the cell cycle and inhibit protein synthesis. Moreover, depending on the intensity and duration, cold stress can activate the apoptotic program, or lead to necrosis [34]. Thus, it is conceivable that prolonged exposure to cold temperature could interfere with the myogenic properties of SCs. Nevertheless, we found that the brief period of exposure to ice-cold temperature did not interfere with the ability of SCs to proliferate and differentiate in vitro or with their in vivo regeneration potential, suggesting that ICT does not alter SC function. Indeed, SCs isolated by ICT behaved similarly to those isolated using the pre-plating method or the magnetic bead isolation kit. Of note, both the magnetic bead and the FACS sorting isolation methods involve far lengthier incubation times on ice compared to our method without interfering with SC behavior and function in vitro and in vivo [16, 17, 21, 22]. Interestingly, Marg A et al. recently found that storing human muscle biopsies at 4 °C, in low serum medium and no O_2_ for up to 35 days and subsequent culture at 37 °C and 21% oxygen led to SCs expansion outside the fiber fragments. Surprisingly, the purity of the outgrowing colonies of SCs was 100% myogenic cells, since non-myogenic cells such as fibroblasts did not survive prolonged storage at hypothermic conditions [33]. Therefore, it is likely that SCs tolerance for cold stress is high compared to other cell types, and this trait can be exploited as we did with our ICT method, to improve the purity of SCs grown in vitro.

Recently, Yoshioka K et al. described an improved version of the pre-plating method, reducing the isolation and purification procedure to 2.5 days in total, while increasing the cell yield, and significantly improving the purity of the resulting SC culture by introducing a re-plating step [15]. The re-plating step performed at day 2.5 of culture involves the detachment of all adhered cells including fibroblasts and SCs with trypsin, and replating on matrigel coated dishes [15]. While the purity of the resultant SC culture is comparable to ours, our method involves fewer steps in total and only one overnight pre-plating. In addition, the ICT method doubles up as a very gentle passaging technique, allowing long-term serial expansion of SCs ex vivo, without altering their proliferation and differentiation properties. Whereas the FACS sorting method is and will remain the gold standard for the study of SCs immediately after isolation, the ICT approach will likely become the method of choice for the in vitro expansion of SCs. After each ICT passage, the already expanded SCs can be cryopreserved, and stored until needed. With the ICT method we were able to passage proliferating mouse and human SCs for at least 10 times, expanding their number 150- and 300-fold, respectively. This represents a clear advantage over the most commonly used passaging reagent trypsin, which we and others have shown, typically accelerates the differentiation of passaged SCs after only two passages [11]. Apart from being a relatively harsh enzymatic passaging reagent, trypsin leads to the detachment of all the cells in the dish including SCs that are already committed to differentiate into myotubes, as well as any contaminating cells, which might contribute towards the loss of SC proliferative potential and accelerated differentiation. Indeed, even a gentler detachment solution like Accutase leads to loss of myogenic proliferative properties and accelerates differentiation. By contrast, the ICT approach favors the detachment of only the SCs that have not yet committed to differentiate, and in particular the slow dividing population which has previously been shown to retain stemness and long-term self-renewal ability [31]. It is conceivable that being ‘true’ stem cells, the slow dividing SCs detaching in response to cold temperature are the so-called first responders to stress or injury in vivo [5], a hypothesis that will be the subject of future investigation in our laboratory.

In a recent study, Gregory WC et al. demonstrated that hSCs differentiate and lose their proliferative potential when maintained in high mitogen conditions ex vivo. They used inhibition of p38 signalling to prevent the differentiation of SCs and promote their expansion [35]. Using our method, we were able to achieve the same but with minimal manipulation, maintaining the proliferative capacity of hSC ex vivo for an extended period of time, to a similar degree using muscle biopsies taken from a wide range of ages (between 50 and 90 years old). This is an important technical advance for both basic and clinical research since it will allow researchers to obtain sufficient number of cells for transplantation or intervention studies, while reducing the number of human biopsies required. Indeed, a major obstacle to stem cell-based therapies has been the scarcity of human muscle tissue specimens and the limited number of cells that can be obtained for transplantation.

Moreover, successful transplantation requires the use of freshly isolated SCs because culturing and expanding them in vitro greatly reduces their engraftment capacity [13]. Here, we show that SCs isolated and expanded using the ICT method do not lose their regenerative capacity.

Another advantage of the ICT method is the improved longevity of cultured myotubes. Generally, SCs differentiate into myotubes by day 7 of culture and shortly after, begin to detach [11, 12]. Notably, SCs isolated and passaged with the ICT method could be maintained in culture for up to 2 months, even once they have differentiated into myotubes, likely due to the lack of contaminating cells such as fibroblasts. The purity of the isolated SCs is of paramount importance for in vitro studies since even 97% purity is insufficient to prevent overgrowth by non-myogenic cells, as we demonstrated. While important for cell growth, growth factors produced by fibroblasts, have been linked to senescence induction in long term cultures of mesenchymal stem cells [36].

## Conclusions

In the quest for new and improved SC isolation methods, the ideal technique would permit the isolation of pure SCs with minimal manipulation, that can be expanded ex vivo without losing their stemness and regenerative capacity. In terms of purity of the isolated cell population, the ICT method outperforms others such as the pre-plating method or the magnetic beads isolation method. Compared to other commonly used methods, it is fast and easy to perform, and apart from the time required for enzymatic digestion (1.5 h), it involves minimal manipulation of the cells. Finally, using the ICT approach, SCs can be expanded for extended periods of time without losing their proliferation and differentiation potential. This in turn drastically reduces the number of mice or muscle biopsies required to obtain sufficient number of cells.

Overall, the cost-effectiveness, accessibility and technical simplicity of this method, as well as its remarkable efficiency, represent major improvements over existing methods, and will no doubt accelerate SC basic and translational research bringing their therapeutic use closer to the clinic. Finally, this is a proof of concept study, and the ICT method can be further optimised, adapted, and improved for use in different experimental settings.

## Supplementary Information

**Additional file 1: Figure S1.** Myogenic properties of SCs isolated with the ICT method. A. Percent of myogenic colony formation was calculated as percent growing clones out of the total seeded single cells per well (60 per 96-well plate) among ICT- and MB-isolated SCs (n=3 independent experiments). B. Number of cells per clone in single clone-derived ICT- and MB-isolated SCs at 48 and 72 h of culture in GM. ICT SCs, n= 37 clones analyzed per experiment. MB SCs, n= 37 clones analyzed per experiment. C. Percent of ICT- and MB-isolated SCs positive for Pax7 at day 2 of culture in GM. (n=3 independent experiments). D. Percent fusion of single clone-derived ICT and MB-isolated SCs after differentiation (4 days in GM followed by 3 days in DM). Fusion index: number of nuclei within myotubes divided by total number nuclei. E. Number of nuclei per myotube in single clone-derived ICT and MB-isolated SCs after differentiation (4 days in GM followed by 3 days in DM). (n=3 independent experiments). Error bars represent mean ± sem.

**Additional file 2: Figure S2.** Increased longevity in culture of ICT-isolated SCs. A. Representative bright field images of ICT-isolated SCs at day 3, 5, 11 and 17 of culture in DM. B. Representative bright field image of MB isolated SCs at day 3 of culture in DM. C. Total number of days in culture of ICT- and MB-isolated SCs. D. Representative bright field images showing the heterogeneous muscle cell culture after pre-plating, at 3 and 5 days of culture in GM. Error bars represent mean ± sem. *P < 0.05 by Student’s t-test.

**Additional file 3: Figure S3.** In vivo functional validation of SCs isolated using the ICT and MB method. A. Representative immunofluorescence images of dystrophin positive fibers (green) and nuclei (blue) in mdx tibialis muscle at 30 days following intra-muscular injection of 15 x 10^3^ SCs immediately after ICT isolation or after 3 day-expansion in culture following ICT isolation. B. Quantification of the number of dystrophin positive fibers per TA muscle section in mdx mice (ICT, n=5 mdx mice; ICT-expanded, n=4 mdx mice). Scale bar=100μm. Error bars represent mean ± sem. C. Representative immunofluorescence images of dystrophin positive fibers (green) and nuclei (blue) in mdx tibialis muscle at 30 days following intra-muscular injection of 15 x 10^3^ ICT-isolated SCs (left), or MB-freshly isolated SCs (right). B. Quantification of the number of dystrophin positive fibers per TA section in mdx mice (ICT, n=5 mdx mice; MB, n=5 mdx mice). Scale bar=100μm. Error bars represent mean ± sem.

**Additional file 4: Figure S4.** Characterisation of the human muscle-derived cells obtained after pre-plating and prior to ICT. A. Representative bright field images of human muscle-derived cells at day 3 and 10 of culture in GM, and at day 10 of culture in DM. B. Representative immunofluorescence images of the heterogeneous culture of human muscle-derived cells stained for Pax7 (red) and nuclei (blue). Graph shows percentage of cells positive for Pax7 at day 2 of culture in GM. C. Representative immunofluorescence images of the heterogeneous human muscle cell culture stained for myogenin (red) and nuclei (blue). Graph shows percent of cells positive for myogenin at day 5 of culture in GM. D. Representative immunofluorescence images of the heterogeneous culture of human muscle-derived cells stained for MHC (red) and nuclei (blue). Graph shows percent cell fusion after differentiation (10 days in GM followed by 10 days in DM). E. Graph shows percent myogenic cells in the human heterogenous muscle cells obtained after pre-plating or after ICT at day 5 of culture in GM, calculated by IF staining for myogenin (n= 3 independent experiments, 10 images analysed per experiment). Scale bar=100μm. Error bars represent mean ± sem. ****P < 0.0001 by Student’s t-test.

## Data Availability

All data generated or analyzed during this study are included in this published article [and its supplementary information files]. The datasets used and/or analyzed during the current study are available from the corresponding author on reasonable request.

## References

[1] Chang NC, Rudnicki MA. Satellite Cells: The Architects of Skeletal Muscle. Curr Top Dev Biol. 2014;107:161–81. 10.1016/B978-0-12-416022-4.00006-8 24439806

[2] Wang YX, Dumont NA, Rudnicki MA. Muscle stem cells at a glance. J Cell Sci. 2014;127:4543–8. 10.1242/jcs.151209 PMC4215708 25300792

[3] Mauro A. Satellite cell of skeletal muscle fibers. J Biophys Biochem Cytol. 1961;9:493–5. 10.1083/jcb.9.2.493 PMC2225012 13768451

[4] Wang YX, Rudnicki MA. Satellite cells, the engines of muscle repair. Nat Rev Mol Cell Biol. 2012;13:127–33. 10.1038/nrm3265 22186952

[5] Evano B, Tajbakhsh S. Skeletal muscle stem cells in comfort and stress. npj Regen Med. 2018;3:24. 10.1038/s41536-018-0062-3 PMC6303387 30588332

[6] Feige P, Rudnicki MA. Muscle stem cells. Curr Biol. 2018;28:581–98. 10.1016/j.cub.2018.02.064 PMC6953716 29787715

[7] Sambasivan R, Yao R, Kissenpfennig A, van Wittenberghe L, Paldi A, Gayraud-Morel B, et al. Pax7-expressing satellite cells are indispensable for adult skeletal muscle regeneration. Development. 2011;138:3647–56. 10.1242/dev.067587 21828093

[8] Relaix F, Zammit PS. Satellite cells are essential for skeletal muscle regeneration: The cell on the edge returns centre stage. Development (Cambridge). 2012;139:2845–56. 10.1242/dev.069088 22833472

[9] Tedesco FS, Dellavalle A, Diaz-Manera J, Messina G, Cossu G. Repairing skeletal muscle: Regenerative potential of skeletal muscle stem cells. J Clin Investig. 2010;120:11–9. 10.1172/JCI40373 PMC2798695 20051632

[10] Keire P, Shearer A, Shefer G, Yablonka-Reuveni Z. Isolation and culture of skeletal muscle myofibers as a means to analyze satellite cells. Methods Mol Biol. 2013;946:431–68. 10.1007/978-1-62703-128-8_28 PMC3633469 23179849

[11] Danoviz ME, Yablonka-Reuveni Z. Skeletal muscle satellite cells: Background and methods for isolation and analysis in a primary culture system. Methods Mol Biol. 2012;798:21–52. 10.1007/978-1-61779-343-1_2 PMC3325159 22130829

[12] Jonah D, Lee BCS, Lisa M, Larkin KWV. Isolation and Purification of Satellite Cells for Skeletal Muscle Tissue Engineering. J Regen Med. 2015;3:117. 10.4172/2325-9620.1000117 PMC4582791 26413555

[13] Rinaldi F, Perlingeiro RCR. Stem cells for skeletal muscle regeneration: Therapeutic potential and roadblocks. Transl Res. 2014. 10.1016/j.trsl.2013.11.006 PMC3976768 24299739

[14] Gharaibeh B, Lu A, Tebbets J, Zheng B, Feduska J, Crisan M, et al. Isolation of a slowly adhering cell fraction containing stem cells from murine skeletal muscle by the preplate technique. Nat Protoc. 2008;163:409–17. 10.1038/nprot.2008.142 18772878

[15] Yoshioka K, Kitajima Y, Okazaki N, Chiba K, Yonekura A, Ono Y. A Modified Pre-plating Method for High-Yield and High-Purity Muscle Stem Cell Isolation From Human/Mouse Skeletal Muscle Tissues. Front Cell Dev Biol. 2020;8:793. 10.3389/fcell.2020.00793 PMC7438441 32903486

[16] Pasut A, Oleynik P, Rudnicki MA. Isolation of muscle stem cells by fluorescence activated cell sorting cytometry. Methods Mol Biol. 2012;798:53–64. 10.1007/978-1-61779-343-1_3 22130830

[17] Liu L, Cheung TH, Charville GW, Rando TA. Isolation of skeletal muscle stem cells by fluorescence-activated cell sorting. Nat Protoc. 2015;10:1612–24. 10.1038/nprot.2015.110 PMC4793971 26401916

[18] Chapman MR, Balakrishnan KR, Li J, Conboy MJ, Huang H, Mohanty SK, et al. Sorting single satellite cells from individual myofibers reveals heterogeneity in cell-surface markers and myogenic capacity. Integr Biol (United Kingdom). 2013;5:692–702. 10.1039/c3ib20290a PMC4803067 23407661

[19] Fukada SI, Higuchi S, Segawa M, Koda KI, Yamamoto Y, Tsujikawa K, et al. Purification and cell-surface marker characterization of quiescent satellite cells from murine skeletal muscle by a novel monoclonal antibody. Exp Cell Res. 2004;296:245–55. 10.1016/j.yexcr.2004.02.018 15149854

[20] Sherwood RI, Christensen JL, Conboy IM, Conboy MJ, Rando TA, Weissman IL, et al. Isolation of adult mouse myogenic progenitors: Functional heterogeneity of cells within and engrafting skeletal muscle. Cell. 2004;119:543–54. 10.1016/j.cell.2004.10.021 15537543

[21] Montarras D, Morgan J, Collins C. Direct Isolation of Satellite Cells for Skeletal Muscle Regeneration. Mol Cell Biol. 2005;309:2064–7. 10.1126/science.1114758 16141372

[22] Blanco-Bose WE, Yao CC, Kramer RH, Blau HM. Purification of mouse primary myoblasts based on α7 integrin expression. Exp Cell Res. 2001;265:212–20. 10.1006/excr.2001.5191 11302686

[23] Benedetti A, Fiore PF, Madaro L, Lozanoska-Ochser B, Bouché M. Targeting pkcθ promotes satellite cell self-renewal. Int J Mol Sci. 2020;21:2419. 10.3390/ijms21072419 PMC7177808 32244482

[24] Fiore PF, Benedetti A, Sandonà M, Madaro L, de Bardi M, Saccone V, et al. Lack of PKCθ promotes regenerative ability of muscle stem cells in chronic muscle injury. Int J Mol Sci. 2020;21:932. 10.3390/ijms21030932 PMC7037041 32023816

[25] Rizzo G, di Maggio R, Benedetti A, Morroni J, Bouche M, Lozanoska-Ochser B. Splenic Ly6Chi monocytes are critical players in dystrophic muscle injury and repair. JCI Insight. 2020;5:e130807. 10.1172/jci.insight.130807 PMC7098719 31874104

[26] Lozanoska-Ochser B, Benedetti A, Rizzo G, Marrocco V, di Maggio R, Fiore P, et al. Targeting early PKCθ-dependent T-cell infiltration of dystrophic muscle reduces disease severity in a mouse model of muscular dystrophy. J Pathol. 2018;244:323–333. 10.1002/path.5016 29214629

[27] Juliano RL, Gagalang E. The adhesion of Chinese hamster cells. I. Effects of temperature, metabolic inhibitors and proteolytic dissection of cell surface macromolecules. J Cell Physiol. 1977;92:209–20. 10.1002/jcp.1040920209 881433

[28] Rico F, Chu C, Abdulreda MH, Qin Y, Moy VT. Temperature modulation of integrin-mediated cell adhesion. Biophys J. 2010;99:1387–96. 10.1016/j.bpj.2010.06.037 PMC2931747 20816050

[29] Machida S, Spangenburg EE, Booth FW. Primary rat muscle progenitor cells have decreased proliferation and myotube formation during passages. Cell Prolif. 2004;37:267–77. 10.1111/j.1365-2184.2004.00311.x PMC6495777 15245563

[30] Tierney MT, Sacco A. Satellite Cell Heterogeneity in Skeletal Muscle Homeostasis. Trends Cell Biol. 2016;26:434–444. 10.1016/j.tcb.2016.02.004 PMC4877266 26948993

[31] Ono Y, Masuda S, Nam HS, Benezra R, Miyagoe-Suzuki Y, Takeda S. Slow-dividing satellite cells retain long-term self-renewal ability in adult muscle. J Cell Sci. 2012;125:1309–17. 10.1242/jcs.096198 22349695

[32] Charville GW, Cheung TH, Yoo B, Santos PJ, Lee GK, Shrager JB, et al. Ex vivo expansion and in vivo self-renewal of human muscle stem cells. Stem Cell Reports. 2015;5:621–32. 10.1016/j.stemcr.2015.08.004 PMC4624935 26344908

[33] Marg A, Escobar H, Gloy S, Kufeld M, Zacher J, Spuler A, et al. Human satellite cells have regenerative capacity and are genetically manipulable. J Clin Investig. 2014;124:4257–65. 10.1172/JCI63992 PMC4191042 25157816

[34] Sonna L, Fujita J, Gaffin SL, Craig M. Molecular Biology of Thermoregulation Invited Review: Effects of heat and cold stress on mammalian gene expression. J Appl Physiol. 2002;92:1725–42. 10.1152/japplphysiol.01143.2001 11896043

[35] Judson RN, Quarta M, Oudhoff MJ, Soliman H, Yi L, Chang CK, et al. Inhibition of Methyltransferase Setd7 Allows the In Vitro Expansion of Myogenic Stem Cells with Improved Therapeutic Potential. Cell Stem Cell. 2018;22:177–90. 10.1016/j.stem.2017.12.010 PMC6031334 29395054

[36] Ito T, Sawada R, Fujiwara Y, Seyama Y, Tsuchiya T. FGF-2 suppresses cellular senescence of human mesenchymal stem cells by down-regulation of TGF-β2. Biochem Biophys Res Commun. 2007;359:108–14. 10.1016/j.bbrc.2007.05.067 17532297

