# Supplementary figures and images for: A novel approach for the isolation and long-term expansion of pure satellite cells based on ice-cold treatment
Source: Skelet Muscle. 2021 Mar 17;11:7. doi: 10.1186/s13395-021-00261-w (PMC7968259; doi:10.1186/s13395-021-00261-w)

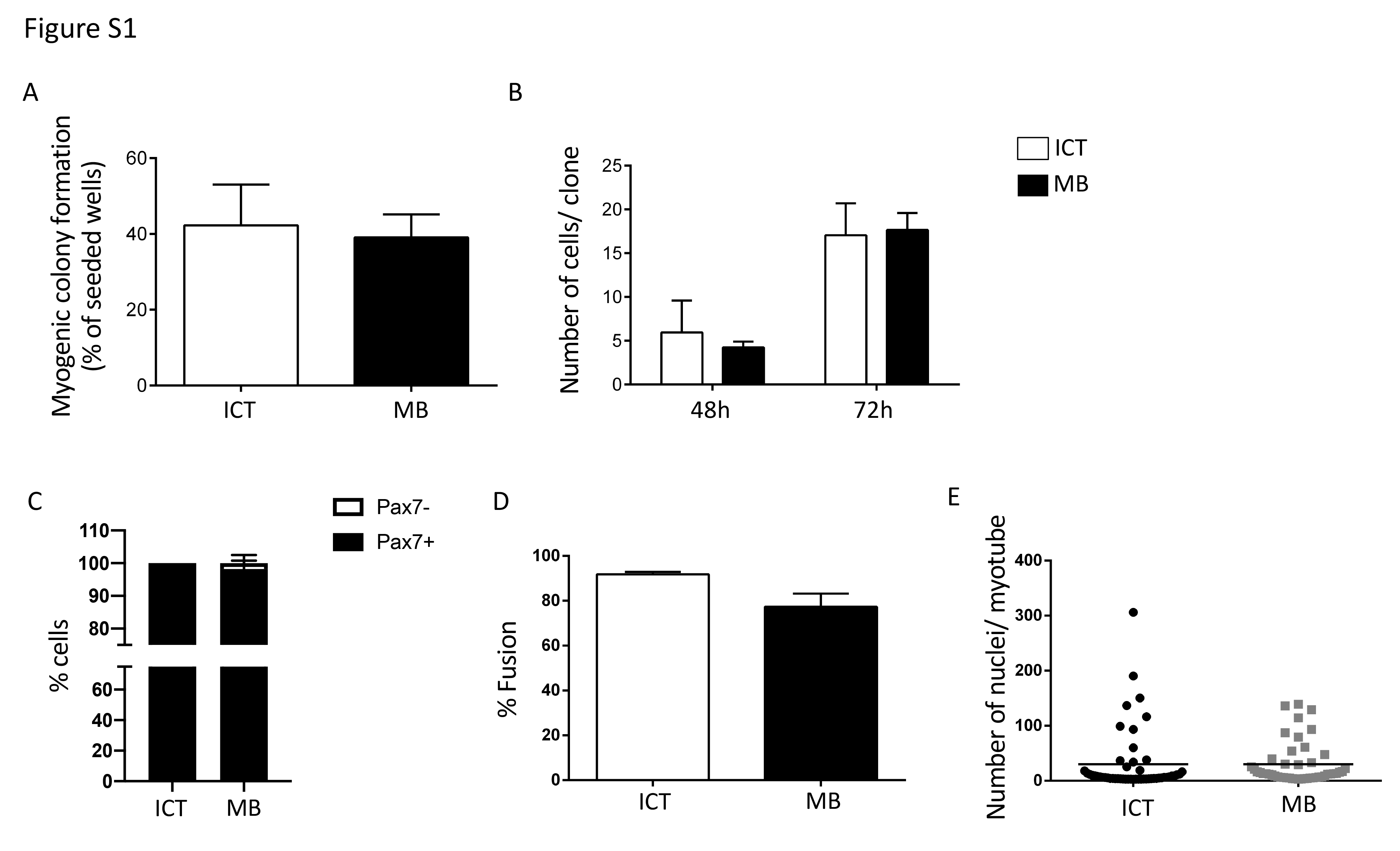

Supplement: Supplementary file 1 — Additional file 1: Figure S1. Myogenic properties of SCs isolated with the ICT method. A. Percent of myogenic colony formation was calculated as percent growing clones out of the total seeded single cells per well (60 per 96-well plate) among ICT- and MB-isolated SCs (n=3 independent experiments). B. Number of cells per clone in single clone-derived ICT- and MB-isolated SCs at 48 and 72 h of culture in GM. ICT SCs, n= 37 clones analyzed per experiment. MB SCs, n= 37 clones analyzed per experiment. C. Percent of ICT- and MB-isolated SCs positive for Pax7 at day 2 of culture in GM. (n=3 independent experiments). D. Percent fusion of single clone-derived ICT and MB-isolated SCs after differentiation (4 days in GM followed by 3 days in DM). Fusion index: number of nuclei within myotubes divided by total number nuclei. E. Number of nuclei per myotube in single clone-derived ICT and MB-isolated SCs after differentiation (4 days in GM followed by 3 days in DM). (n=3 independent experiments). Error bars represent mean ± sem. [file 13395_2021_261_MOESM1_ESM.tif]

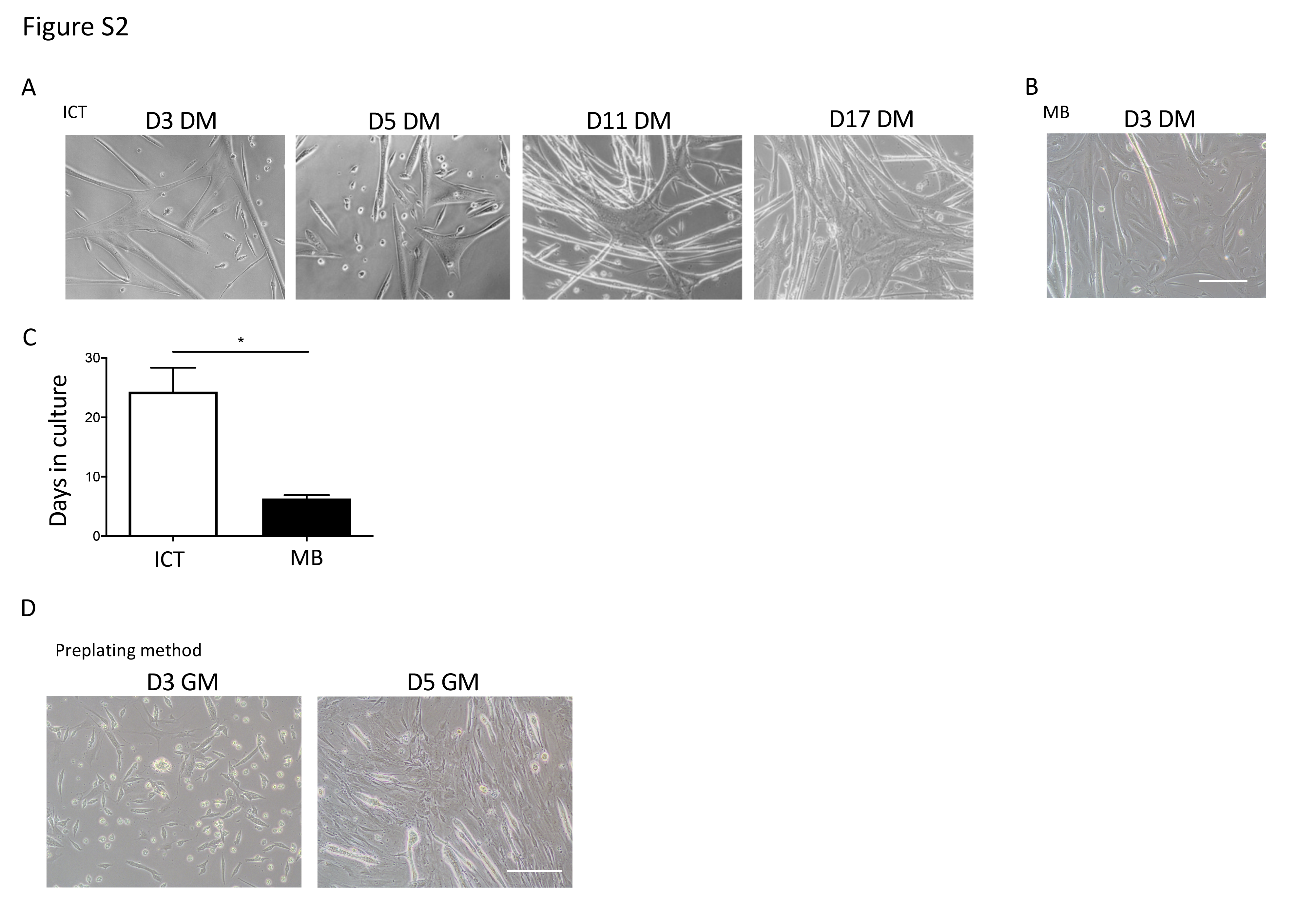

Supplement: Supplementary file 2 — Additional file 2: Figure S2. Increased longevity in culture of ICT-isolated SCs. A. Representative bright field images of ICT-isolated SCs at day 3, 5, 11 and 17 of culture in DM. B. Representative bright field image of MB isolated SCs at day 3 of culture in DM. C. Total number of days in culture of ICT- and MB-isolated SCs. D. Representative bright field images showing the heterogeneous muscle cell culture after pre-plating, at 3 and 5 days of culture in GM. Error bars represent mean ± sem. *P < 0.05 by Student’s t-test. [file 13395_2021_261_MOESM2_ESM.tif]

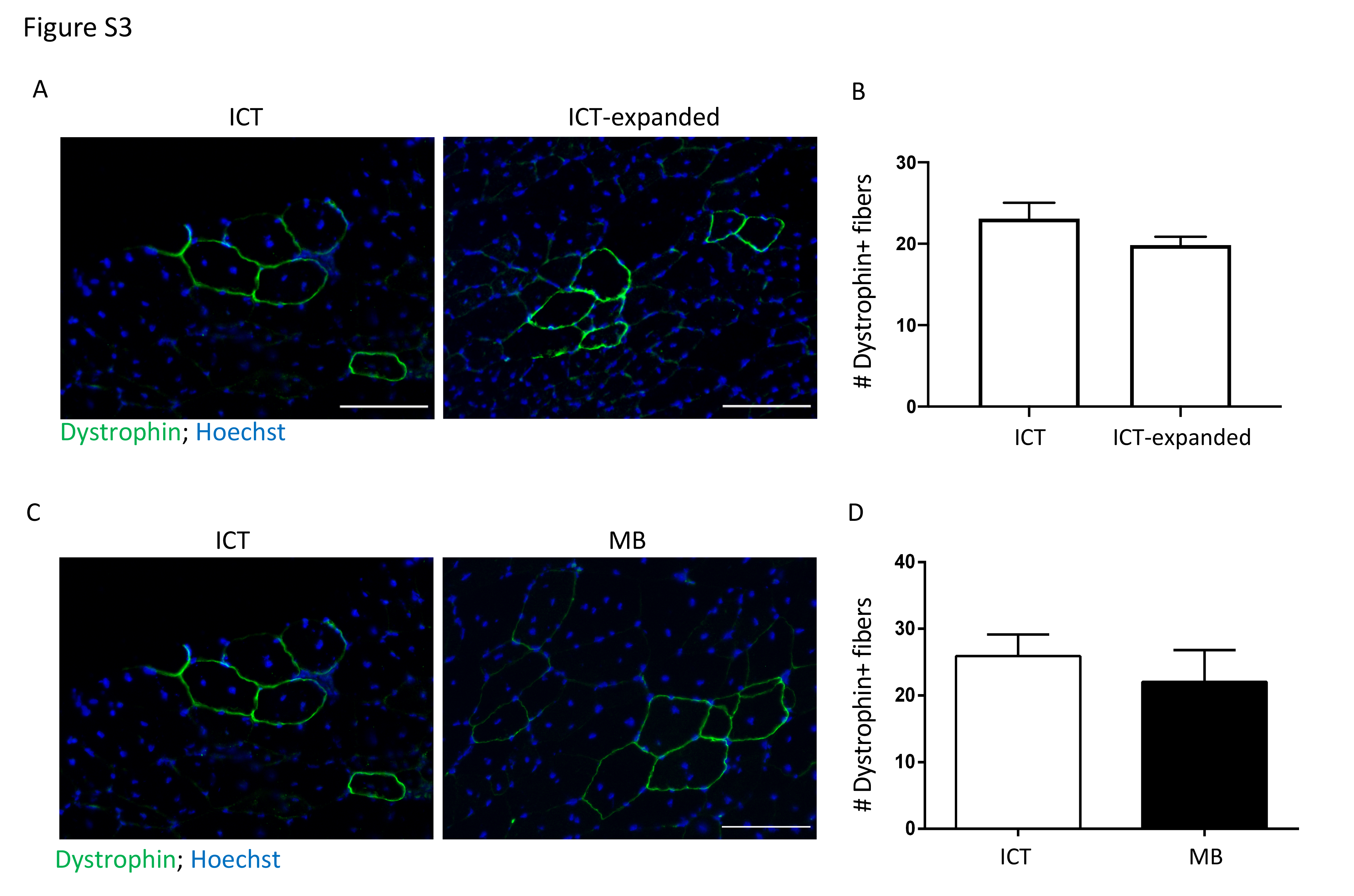

Supplement: Supplementary file 3 — Additional file 3: Figure S3. In vivo functional validation of SCs isolated using the ICT and MB method. A. Representative immunofluorescence images of dystrophin positive fibers (green) and nuclei (blue) in mdx tibialis muscle at 30 days following intra-muscular injection of 15 x 103 SCs immediately after ICT isolation or after 3 day-expansion in culture following ICT isolation. B. Quantification of the number of dystrophin positive fibers per TA muscle section in mdx mice (ICT, n=5 mdx mice; ICT-expanded, n=4 mdx mice). Scale bar=100μm. Error bars represent mean ± sem. C. Representative immunofluorescence images of dystrophin positive fibers (green) and nuclei (blue) in mdx tibialis muscle at 30 days following intra-muscular injection of 15 x 103 ICT-isolated SCs (left), or MB-freshly isolated SCs (right). B. Quantification of the number of dystrophin positive fibers per TA section in mdx mice (ICT, n=5 mdx mice; MB, n=5 mdx mice). Scale bar=100μm. Error bars represent mean ± sem. [file 13395_2021_261_MOESM3_ESM.tif]

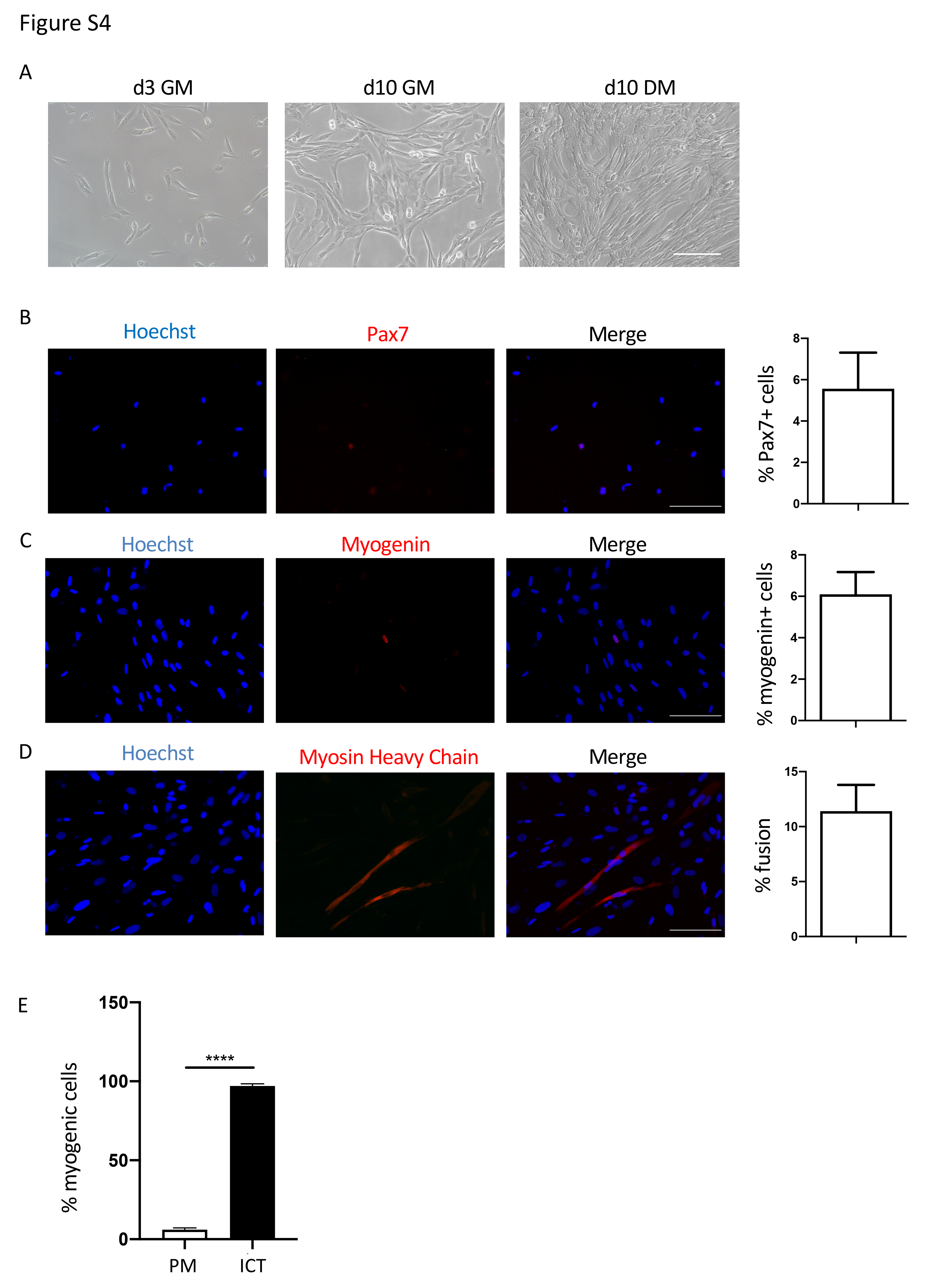

Supplement: Supplementary file 4 — Additional file 4: Figure S4. Characterisation of the human muscle-derived cells obtained after pre-plating and prior to ICT. A. Representative bright field images of human muscle-derived cells at day 3 and 10 of culture in GM, and at day 10 of culture in DM. B. Representative immunofluorescence images of the heterogeneous culture of human muscle-derived cells stained for Pax7 (red) and nuclei (blue). Graph shows percentage of cells positive for Pax7 at day 2 of culture in GM. C. Representative immunofluorescence images of the heterogeneous human muscle cell culture stained for myogenin (red) and nuclei (blue). Graph shows percent of cells positive for myogenin at day 5 of culture in GM. D. Representative immunofluorescence images of the heterogeneous culture of human muscle-derived cells stained for MHC (red) and nuclei (blue). Graph shows percent cell fusion after differentiation (10 days in GM followed by 10 days in DM). E. Graph shows percent myogenic cells in the human heterogenous muscle cells obtained after pre-plating or after ICT at day 5 of culture in GM, calculated by IF staining for myogenin (n= 3 independent experiments, 10 images analysed per experiment). Scale bar=100μm. Error bars represent mean ± sem. ****P < 0.0001 by Student’s t-test. [file 13395_2021_261_MOESM4_ESM.tif]
